# *Streptococcus pneumoniae* carriage in adults during the COVID-19 pandemic in Portugal: dominance of serotypes included in broader PCVs and of serotype 3

**DOI:** 10.1128/msphere.00082-25

**Published:** 2025-06-10

**Authors:** Sónia T. Almeida, A. Cristina Paulo, Alexandra S. Simões, Bárbara Ferreira, Raquel Sá-Leão

**Affiliations:** 1 Laboratory of Molecular Microbiology of Human Pathogens, Instituto de Tecnologia Química e Biológica António Xavier, Universidade Nova de Lisboa 98819 , Oeiras, Lisbon, Portugal; University of Napoli Federico II, Naples, Italy

**Keywords:** *Streptococcus pneumoniae*, carriage, adults, qPCR, serotype, risk factors

## Abstract

*Streptococcus pneumoniae* (pneumococcus) is a leading cause of infections, particularly in infants and the elderly. Recent advances in molecular methods suggest higher pneumococcal carriage rates among adults than previously estimated, raising questions about their role in transmission. This study aimed to estimate pneumococcal carriage prevalence, identify circulating serotypes, and assess risk factors for colonization among adults during the COVID-19 pandemic in Portugal. We conducted a prospective observational study among civil servants aged ≥18 years in Oeiras Municipality from February 2021 to February 2022. Paired nasopharyngeal and oropharyngeal samples were analyzed using qPCR to detect pneumococcal carriage and 66 serotypes/serogroups. This included novel primers and probes for serotypes 4 and 24B/F, overcoming previous concerns associated with false positivity. Risk factors were identified using Bayesian adaptive sampling for variable selection in generalized linear model. Among 3,574 participants, 6.9% were pneumococcal carriers through qPCR without prior culture enrichment. Carriage rates were higher in oropharyngeal than nasopharyngeal samples (5.3% vs 3.7%, *P* < 0.001). Twenty-six serotypes/serogroups were identified, with the most common being non-encapsulated (NT), 10A, 23B, 3, 11A/D, 33A/F/37, 16F, and 31. Excluding NT, the most frequent serotypes collectively accounted for 45.3% of all carriers. Vaccine coverage estimates were 13.5% for PCV13, 20.4% for PCV15, 40.0% for PCV20, and 64.1% for PCV21. Contact with children < 18 years increased the odds of colonization by 2.73-fold (95% confidence interval [CI], 2.01–3.75), while being male reduced the odds by 54% (odds ratio = 0.46; 95% CI, 0.30–0.69). These findings emphasize the need for ongoing surveillance to clarify adults’ role in pneumococcal transmission and support prevention strategies, including adult vaccination and community-level interventions, to mitigate pneumococcal disease.

**IMPORTANCE:**

*Streptococcus pneumoniae* is a major pathogen causing significant disease worldwide, yet adult carriage remains underexplored. By evaluating pneumococcal carriage among adults in Portugal during the COVID-19 pandemic, this study provides critical insights into circulating serotypes, including those not targeted by 13-valent pneumococcal conjugate vaccine (PCV13), and highlights key risk factors such as contact with children and sex differences. The findings reveal substantial potential coverage for newer PCVs. This work underscores the importance of adult-focused prevention strategies, including vaccination and ongoing surveillance, to reduce pneumococcal transmission and disease burden in the community.

## INTRODUCTION

*Streptococcus pneumoniae* (or pneumococcus) is an important inhabitant of the human upper respiratory tract that is known to be responsible for bacterial infections worldwide, such as otitis media, pneumonia, bacteremia, and meningitis (1, 2).

To reduce the burden of pneumococcal disease, pneumococcal vaccines, targeting a subset of the more than 100 known pneumococcal capsular types (or serotypes), have been developed (3). In Europe, pneumococcal conjugate vaccines (PCVs) covering 10, 13, 15, and 20 serotypes are available, alongside the 23-valent pneumococcal polysaccharide vaccine (PPSV23). All these vaccines are approved for adult immunization (4). In Portugal, PCV13 was included in the national childhood immunization program in mid-2015. In December 2024, PCV20 replaced PCV13 for children at 2, 4, and 12 months of age. For adults aged ≥ 18 years with specific medical conditions, a sequential vaccination strategy with PCV20 followed by PPSV23 is recommended and provided free of charge. For individuals aged ≥ 65 years without additional risk factors, PPSV23 is recommended with partial reimbursement. Both vaccines are also available in community pharmacies with a 37% reimbursement upon medical prescription (5). PCV21 has been recently approved in the United States (6) and is expected to become available in Europe during 2025.

In Portugal, vaccine coverage among children is consistently high. In 2019, before the onset of COVID-19, PCV13 coverage at 2 years of age was 98.2%, during the COVID-19 pandemic was 98.1% (2020) and 98.0% (2021), decreasing slightly to 97.8% in 2022. This suggests that herd immunity likely played a role in adult *S. pneumoniae* carriage (7).

Pneumococcal disease and transmission are preceded by asymptomatic colonization of the upper respiratory tract (8). Young children, often considered the main reservoir in the community, exhibit high colonization rates frequently exceeding 50% (9–11). Carriage among adults, relying on the culture of nasopharyngeal and/or oropharyngeal samples as recommended by the World Health Organization, is lower, ranging from 1 to 10% (12–14). However, molecular methods with high sensitivity have revealed higher carriage rates in adults, particularly in polymicrobial samples such as the oropharynx, where pneumococci are often present at low density. These approaches suggest pneumococcal carriage in adults of all ages is relatively frequent, 20–40% significantly higher than culture-based estimates (15–19). For example, a 6-month longitudinal study, conducted by our group, among immunocompetent adults aged 25–50 years old, revealed an annual pneumococcal acquisition risk of 57.5%, and a median duration of carriage of 7 weeks, much longer than previously estimated (16).

These findings challenged the traditional understanding of pneumococcal colonization in adults and emphasized the need for further studies to understand the contribution of adults to pneumococcal transmission and disease. Such research is essential for surveillance efforts evaluating the impact of new PCVs and for developing more effective disease prevention strategies.

In this study, we aimed to investigate pneumococcal colonization patterns among adults living in Portugal during the COVID-19 pandemic. Specifically, we estimated the prevalence of pneumococcal carriage, identified serotypes, and examined risk factors associated with pneumococcal colonization in this population.

## RESULTS

### Characteristics of the population

The characteristics of the population are described in Table 1. In short, 3,574 paired nasopharyngeal and oropharyngeal samples were obtained. The mean age of the participants was 43.8 ± 13.2 (range: 18–84 years old). Females were overrepresented (79.4%). More than half (58.3%) of the participants had contact with children; 50.9% had daily interactions.

**TABLE 1:** Socio-demographic and clinical characteristics of the participants by pneumococcal carriage status

Variable	*n* (%)			*P* value^*a*^
	Overall (*N* = 3,574)	Pneumococcal carriage		
		No (*N* = 3,329)	Yes (*N* = 245)	
Gender				**<0.001**
Female	2,837 (79.4)	2,622 (78.7)	218 (89.0)	
Male	737 (20.6)	710 (21.3)	27 (11.0)	
Age (years)^*b*^	43.8 ± 13.2	44.0 ± 13.3	41.1 ± 12.1	**<0.001**
Fasting duration (h)				0.164
<1	933 (26.1)	875 (26.3)	58 (23.7)	
Between 1 and 2	1,538 (43.0)	1,440 (43.3)	98 (40.0)	
>2	1,103 (30.9)	1,014 (30.5)	89 (36.3)	
Mask use (h/day)				0.717
<4	226 (6.3)	211 (6.3)	15 (6.1)	
Between 4 and 8	1,338 (37.4)	1,252 (37.6)	86 (35.1)	
>8	2,010 (56.2)	1,866 (56.1)	144 (58.8)	
Contact children <18 years	2,084 (58.3)	1,891 (56.8)	192 (78.4)	**<0.001**
Frequency of contact with children (days/week)^*c*^				0.370
Daily	1,818 (50.9)	1,644 (49.4)	174 (71.0)	
≥1 but not daily	196 (5.5)	182 (5.5)	14 (5.7)	
<1	70 (2.0)	66 (2.0)	4 (1.6)	
Smoker	866 (24.2)	806 (24.2)	60 (24.5)	0.938
Chronic diseases^*d*^	886 (24.8)	837 (25.1)	49 (20.0)	0.078
Symptoms at time of sampling^*e*^	71 (2.0)	66 (2.0)	5 (2.0)	0.815
Influenza vaccination	960 (26.9)	909 (27.3)	51 (20.8)	**0.030**
Pneumococcal vaccination	88 (2.5)	87 (2.6)	1 (0.4)	**0.029**
Pneumococcal vaccine				>0.999
PPV23	15 (0.4)	15 (0.5)	0 (0.0)	
PCV13	39 (1.1)	39 (1.2)	0 (0.0)	
PCV13/PPV23	5 (0.1)	5 (0.2)	0 (0.0)	
Unknown	29 (0.8)	28 (0.8)	1 (0.4)	
COVID-19 vaccination	2,205 (61.7)	2,071 (62.2)	134 (54.7)	**0.021**
No. of COVID-19 vaccine doses				**0.018**
1	614 (17.2)	583 (17.5)	31 (12.7)	
2	1,310 (36.7)	1,218 (36.6)	92 (37.6)	
3	272 (7.6)	261 (7.8)	11 (4.5)	
Unknown	9 (0.3)	9 (0.3)	0 (0.0)	
Recent antibiotic consumption				0.277
At sampling	21 (0.6)	19 (0.6)	2 (0.8)	
<1 month	80 (2.2)	77 (2.3)	3 (1.2)	
COVID-19 infection at time of sampling	97 (2.7)	90 (2.7)	7 (2.9)	0.838
Past COVID-19 infection	623 (17.4)	580 (17.4)	43 (17.6)	0.931
Time since last COVID-19 infection (months)				0.782
<1	13 (0.4)	13 (0.4)	0 (0.0)	
Between 1 and 3	34 (1.0)	31 (0.9)	3 (1.2)	
>3	575 (16.1)	536 (16.1)	39 (15.9)	

^*a*^ Fisher´s exact test. Values in bold indicate statistical significance.

^*b*^ Mean ± SD, Student’s *t*-test.

^*c*^ Contact was defined as spending time with the child on shared space and physical proximity.

^*d*^ Includes asthma, diabetes, cardiovascular disease, renal disease, hypertension, chronic obstructive pulmonary disease, obesity (see Table S2).

^*e*^ Includes fatigue, sore throat, chest pain, fever, muscle pain, shortness of breath, cough, smell and/or taste loss, and diarrhea (see Table S3).

Regarding medical history, one-quarter of the population had at least one chronic disease (Table 1), with hypertension being the most reported (16.5%) (Table S2); 17.4% reported a past COVID-19 infection (Table 1). Among the few participants (2.0%) who reported symptoms at the time of the sampling, cough and sore throat were the ones most frequently identified (Table S2).

More than half (61.7%) of the participants had received at least one dose of the COVID-19 vaccine, 26.9% had received the seasonal flu vaccine, and only 2.5% had received a pneumococcal vaccine (either PCV13 or PPV23). Antibiotic consumption within the last month was low (2.8%), with 0.6% of the participants reporting antibiotic treatment at the time of the sampling (Table 1).

### Pneumococcal carriage

**Fig 1:**
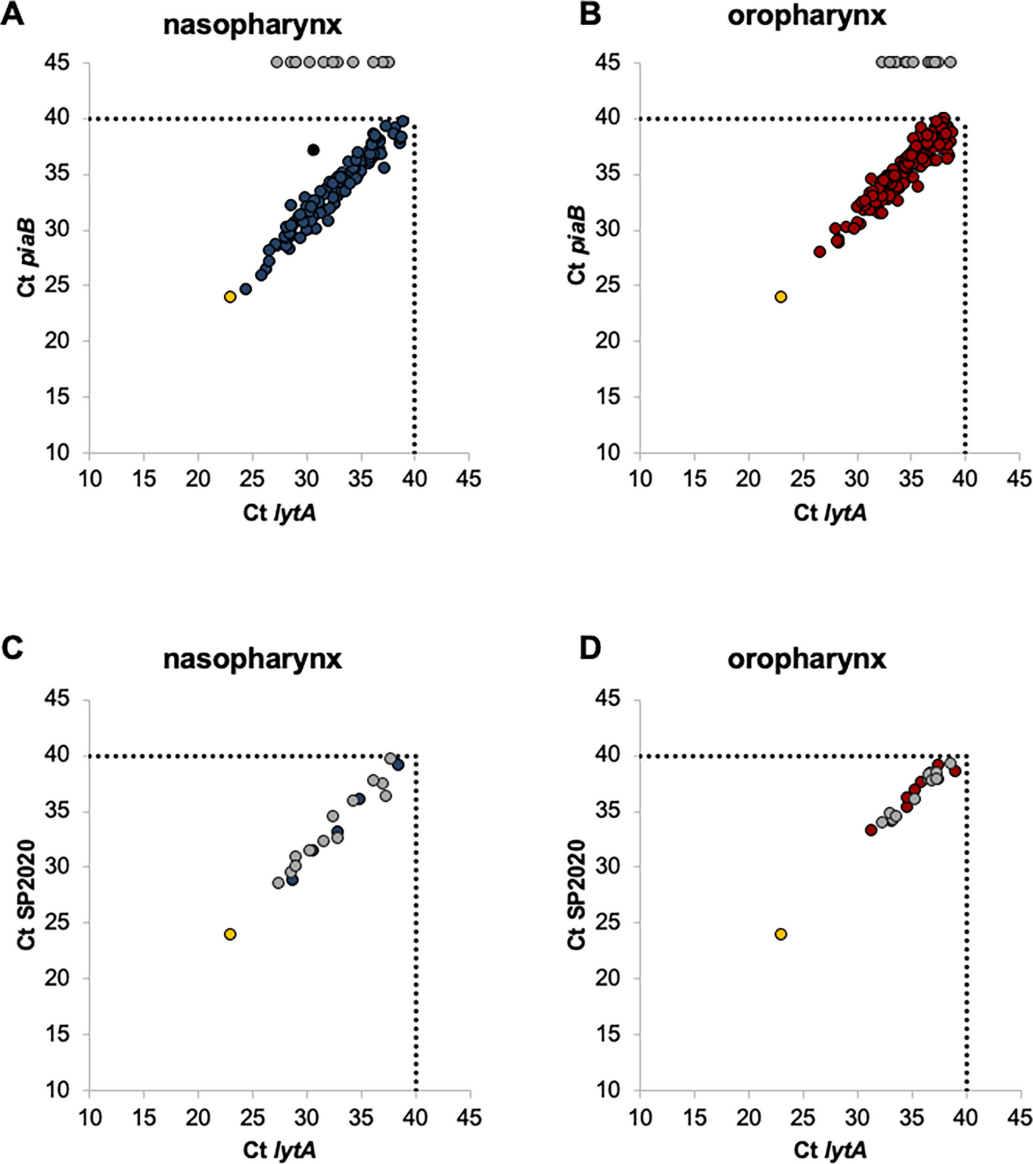
Detection of *S. pneumoniae* by real-time PCR in nasopharyngeal and oropharyngeal samples from adults over 18 years old. (**A, B**) Detection of *S. pneumoniae* using *lytA* and *piaB* targets. (**C, D**) Detection of *S. pneumoniae* using *lytA* and SP2020 targets. Panels C and D include only samples that were *lytA* positive but *piaB* negative or with a Ct difference between *lytA* and *piaB* exceeding 2. Each circle denotes an individual sample, and its position corresponds to the cycle threshold (Ct) values obtained for each gene. Blue circles, nasopharyngeal samples (positive for both genes); red circles, oropharyngeal samples (positive for both genes); gray circles, samples positive for *lytA* but negative for *piaB* in panels A and B, and positive for SP2020 in panels C and D; black circle, excluded sample; yellow circles, positive control (*S. pneumoniae* TIGR4). Dashed lines indicate the Ct value defined to discriminate between positive and negative assays.

We evaluated pneumococcal carriage by qPCR targeting *lytA* and *piaB* genes in nasopharyngeal and oropharyngeal samples from community-dwelling adults. We identified 322 samples positive for *lytA*, of which 90.7% (*n* = 292) were also positive for *piaB*; 282 samples had concordant Cts between *lytA* and *piaB* and were considered positive for pneumococci. The 10 samples with discordant Cts plus 30 samples only positive for *lytA* were tested for the presence of SP2020. All were positive with concordant Cts between SP2020 and *lytA* and were also considered positive for pneumococci (Fig. 1). In total, 322 samples (190 from the oropharynx and 132 from the nasopharynx) were identified as positive for pneumococci (Table 2).

**TABLE 2:** Detection of *S. pneumoniae* carriers according to sampling site^*a*^

Participants, *n*	Sampling site			Carriers, *n* (%)
	Oropharynx, *n* (%)	Nasopharynx, *n* (%)	*P* value	
3,574	190 (5.3%)	132 (3.7%)	**<0.001**	245 (6.9%)

^*a*^ *P* value determined using McNemar’s chi-squared test for paired individuals. Values in bold indicate statistical significance.

The geometric mean of Ct values for *lytA* and *piaB* was significantly lower in positive nasopharyngeal samples than in positive oropharyngeal samples (*P* < 0.001 for both *lytA* and *piaB*), indicating that the density of pneumococci was higher in nasopharyngeal samples than in oropharyngeal samples (Fig. 1 and Table S3). By contrast, *S. pneumoniae* was more frequently detected in oropharyngeal samples than in nasopharyngeal samples: 5.3% vs 3.7% (*P* < 0.001). In 77 (2.2%) participants, pneumococci were detected in both sampling sites. Sampling both nasopharynx and oropharynx significantly increased the detection of pneumococci (*n* = 132 nasopharyngeal samples vs *n* = 245 nasopharyngeal and oropharyngeal samples [*P* < 0.001]; *n* = 190 oropharyngeal samples vs *n* = 245 nasopharyngeal and oropharyngeal samples [*P* < 0.001]). Overall, pneumococcal carriage was detected in 6.9% (*n* = 245) of the participants (Table 2).

### Molecular detection of *S. pneumoniae* serotypes

Pneumococcal positive samples were screened by qPCR targeting 66 serotypes. No positive samples were identified for serotypes/serogroups 1, 2, 6B/D, 7B/C, 10B, 12A/B/F/44/46, 13, 14, 15A/F, 18A/B/C/F, 20, 22A, 28A/F, 34, 35A, and 38. To look for potential false-positive results, we also screened 600 random pneumococcal negative samples (300 from the nasopharynx and 300 from the oropharynx) using primers and probes targeting all serotypes detected among the pneumococcal positive samples. False-positive results were obtained for serotypes 5, 11B/C, 24A, and 39 in both nasopharyngeal and oropharyngeal samples, and for serotypes 9N/L, 17F, and 35B exclusively in oropharyngeal samples (Table S1). Consequently, results for these serotypes were considered unreliable for the sampling site where false positives were detected and were excluded. After the application of these criteria, we assigned a total of 26 distinct capsular types (including NT) to pneumococcal positive samples (Table 3, Fig. S1 and Fig. S2).

**TABLE 3:** Serotype distribution and vaccination potential coverage among pneumococcal carriers

Serotype/serogroup	Pneumococcal carriers % (*n*)
PCV13 serotypes and 6C	
3	6.9 (17)
4	0.4 (1)
6A	0.8 (2)
6C	0.8 (2)
7A/F	1.2 (3)
9A/V	1.6 (4)
19A	0.4 (1)
19F	0.8 (2)
23F	0.4 (1)
PCV15 additional serotypes	
22F	0.8 (2)
33A/F/37	6.1 (15)
Serotypes common to both PCV20/PCV21	
8	4.9 (12)
10A	7.3 (18)
11A/D	6.1 (15)
15B/C	1.2 (3)
PCV21 exclusive serotypes	
9 N/L^*a*^	0.8 (2)
16F	5.7 (14)
17F^*a*^	2.0 (5)
23A	2.4 (6)
23B	7.3 (18)
24B/F	0.4 (1)
31	5.7 (14)
35B^*a*^	2.9 (7)
Other serotypes	
21	2.0 (5)
35F/47F	2.4 (6)
NT	8.6 (21)
No serotype assigned^*b*^	22.9 (56)
Potential coverage^*c*^	
PCV13	13.5%
PCV15	20.4%
PCV20	40.0%
PCV21	64.1%

^*a*^ False-positive results were obtained when pools of oropharyngeal samples (but no of nasopharyngeal samples) negative for pneumococci were tested (see also Table S1). This led to the exclusion of oropharyngeal samples positive for pneumococci with positive results for serotypes 9N/L (*n* = 5), 17F (*n* = 1), and 35B (*n* = 11).

^*b*^ Includes 38 carriers that were negative for all serotypes tested and 18 carriers for whom the only serotype assigned gives false-positive results in pneumococcal negative samples (see main text for details). Serotypes/serogroups tested for which no positive results were obtained: 1, 2, 7B/C, 10B, 12A/B/F/44/46, 13, 14, 15A/F, 18A/B/C/F, 20, 22A, 28A/F, 34, 35A, and 38. Serotypes/serogroups for which false-positive results were obtained in pneumococcal negative pools from oropharyngeal and nasopharyngeal samples: 5, 11B/C, and 39. Serotypes/serogroups for which false-positive results were obtained in pneumococcal negative pools from oropharyngeal samples only: 9N/L, 17F, and 35B.

^*c*^ To estimate the potential coverage of PCVs, serotype 6C was included with PCV13 serotypes.

**Fig 2:**
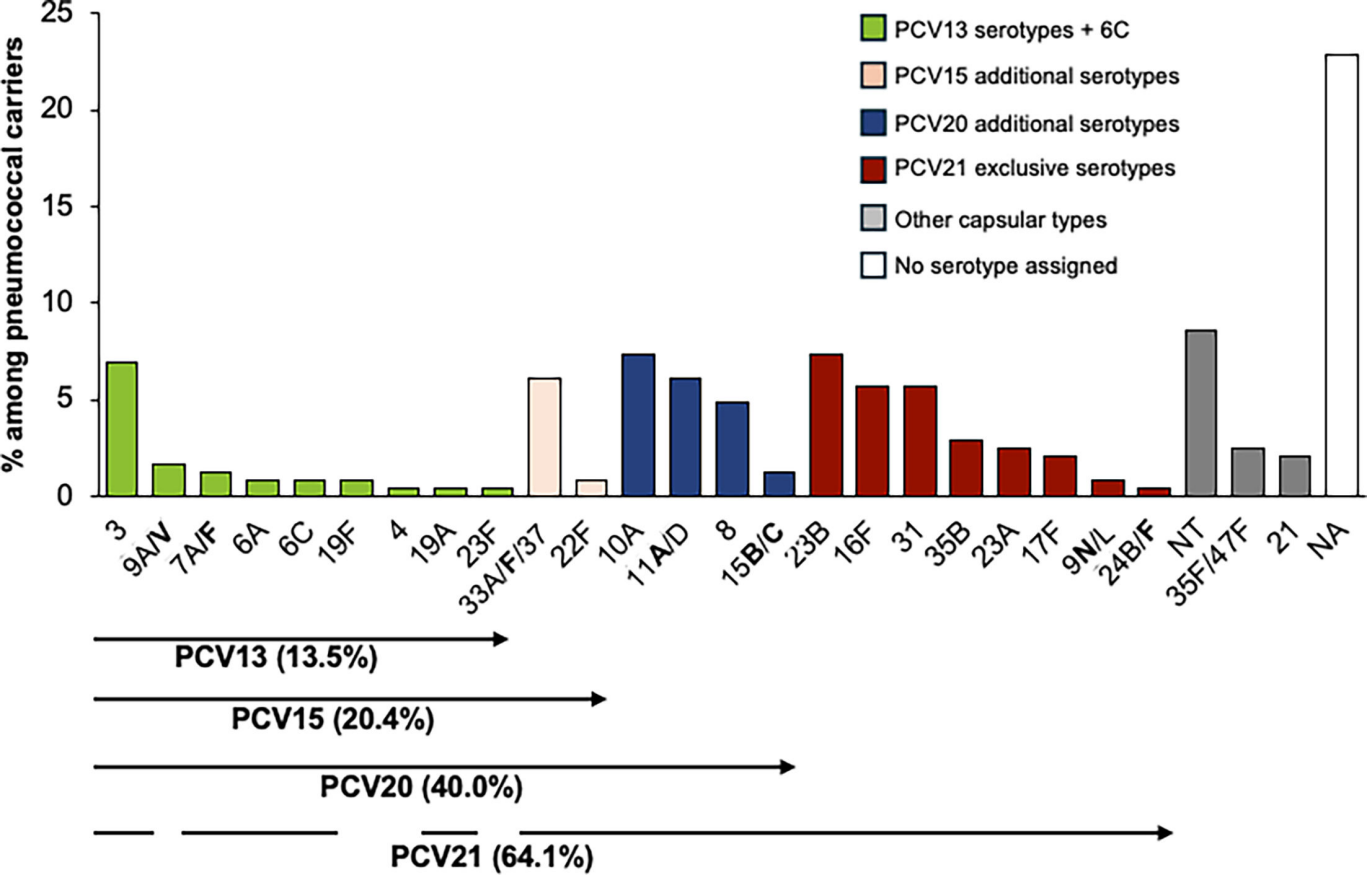
Serotype distribution among pneumococcal carriers. Serotypes in bold are targeted by one or more pneumococcal conjugate vaccines (PCVs). Molecular serotyping was unable to differentiate certain capsular types, which are therefore grouped together (e.g., 33A, 33F, and 37). The numbers in parentheses represent potential vaccine coverage, calculated under the most favorable assumption—for instance, treating all 33A/F/37 as 33F, which is targeted by PCV15, PCV20, and PCV21. Green, PCV13 serotypes plus 6C; pink, PCV15 additional serotypes; blue, PCV20 additional serotypes; red, PCV21 exclusive serotypes; and gray, other capsular types. NA, no serotype assigned; NT, non-encapsulated strains.

Overall, among the 245 pneumococcal carriers, 182 (74.3% of all carriers) had a single serotype assigned, 6 (2.4%) had two serotypes, and 1 had three (0.4%) serotypes. Thirty-seven carriers (15.1%) were negative for all serotypes tested, and 19 (7.8%) yielded potentially false-positive results, and thus no serotype assignment was made (Fig. S2). Among the 77 individuals identified as pneumococcal carriers at both sampling sites, 59 carried the same serotype at both sites. Of the remaining individuals, 12 had no serotype detected, and in 6, a serotype was detected only in the nasopharynx. Combining nasopharyngeal and oropharyngeal did not result in a higher diversity of serotypes detected (Gini-Simpson index of diversity [GSID] = 0.933, 95% confidence interval [CI]: 0.924–0.942) compared to sampling each site individually (GSID = 0.944, 95% CI: 0.931–0.957 for nasopharynx and GSID = 0.919, 95% CI: 0.905–0.933 for oropharynx). Serotypes 3, 10A, 23B, and 33A/F/37 were more frequently detected in oropharyngeal samples compared to nasopharyngeal samples. However, only serotypes 10A (*P* = 0.027) and 33A/F/37 (*P* = 0.003) exhibited statistically significant differences in prevalence between the two sites. In contrast, serotypes 19F (*n* = 2) and 24B/F (*n* = 1) were exclusively identified in nasopharyngeal samples. Additionally, serotypes 6A (*n* = 2) and 19A (*n* = 1) were only found in oropharyngeal samples (Fig. S2). Overall, the most frequently detected serotypes/serogroups were NT (8.6%), 10A (7.3%), 23B (7.3%), 3 (6.9%), 11A/D (6.1%), 33A/F/37 (6.1%), 16F (5.7%), and 31 (5.7%) collectively accounting for 53.9% of all carriers (Fig. 2; Table 3).

PCV13 serotypes (including serotype 6C) were detected in 13.5% of the carriers. The most prevalent PCV13 serotype was 3 (6.9% of all carriers), which accounted for more than half of the PCV13 pneumococci carried by adults. Carriage of non-PCV13 serotypes was 66.9%. Serotypes 22F and 33A/F/37 included in both PCV15 and PCV20 (but not in PCV13) were detected in 6.9% of the carriers. Serotypes 8, 10A, 11A/D, and 15B/C, included in PCV20 and PCV21 (but not in PCV13 or PCV15), were detected in 19.6% of the carriers. Additionally, serotypes 9N/L, 16F, 17F, 23A, 23B, 24B/F, 31, and 35B, which are exclusively included in PCV21, were detected in more than one-quarter (27.3%) of the carriers. Overall, among the 245 carriers, the potential coverage of PCV13, PCV15, PCV20, and PCV21 was 13.5%, 20.4%, 40.0%, and 64.1%, respectively (Fig. 2, Table 3).

### Risk factors for pneumococcal carriage

In the univariable analysis, factors such as gender, age, contact with children < 18 years old, influenza vaccination, pneumococcal vaccination, COVID-19 vaccination, and the number of COVID-19 vaccine doses were found to be associated with pneumococcal carriage (Table 1). These factors were subsequently included in the multivariate generalized linear model (GLM) model with a logit link function (Table 4). Additionally, hypertension, which was linked to pneumococcal carriage, and the symptoms of smell or taste loss—though reported by only five individuals—were also included in the GLM model (Table S2).

**TABLE 4:** Risk factors associated with pneumococcal carriage

Variable	Logistic regression					Bayesian method with g-prior	
	Univariable OR	Multivariable					
		OR	95% CI	*P* value	VIF^*a*^	OR^*b*^	95% CI
Gender, male	0.46	**0.43^*c*^**	**0.28–0.64**	**<0.001**	**1.0**	**0.46**	**0.31–0.69**
Age (years)	0.98	0.99	0.98–1.01	0.33	1.2	–	–
Contact with children <18 years	2.75	**2.57**	**1.89–3.57**	**<0.001**	**1.0**	**2.73**	**2.00–3.74**
Influenza vaccination	0.70	0.88	0.62–1.23	0.46	1.1	–	–
Pneumococcal vaccination	0.15	0.21	0.01–0.98	0.047	1.0	–	–
COVID-19 vaccination	0.73	0.78	0.59–1.04	0.088	1.1	–	–
Smell/taste loss	9.12	9.98	1.20–68.3	0.035	1.0	–	–
Hypertension	0.64	0.65	0.35–1.20	0.16	2.2	–	–

^*a*^ VIF, variance inflation factor.

^*b*^ “–” indicates that the Bayesian method with g-prior was not applied as the variable did not reach statistical sgnificance in the multivariate analysis.

^*c*^ Values in bold indicate statistical significance.

In the multivariable analysis, gender (odds ratio [OR], 0.43 [95% CI, 0.28–0.64]; *P* < 0.001), contact with children < 18 years old (OR, 2.57 [95% CI, 1.89–3.57]; *P* < 0.001), pneumococcal vaccination (OR, 0.21 [95% CI, 0.01–0.98]; *P* = 0.047), and smell/taste loss (OR, 9.98 [95% CI, 1.20–68.3]; *P* = 0.035) were still associated with pneumococcal carriage (Table 4). Of note, the very large confidence interval and the extremely high OR for the variable smell/taste loss are consequences of the sparse data bias, which cannot be corrected with maximum likelihood estimates as used in GLM.

To improve the confidence in the predictors of the model, we used Bayesian adaptive sampling for variable selection in the GLM model with a logit link function. This approach involved 10,000 MCMC iterations in 2^8^ possible models. Estimates for the posterior coefficients under the highest Bayesian probabilistic model were inferred. In this analysis, contact with children < 18 years old significantly increased the odds of being a pneumococcal carrier (OR, 2.73 [95% CI, 2.01–3.75]). Conversely, being male decreased the odds of being a pneumococcal carrier by about 54% (OR, 0.46 [95% CI, 0.30–0.69]).

## DISCUSSION

This study evaluated the prevalence of nasopharyngeal and oropharyngeal pneumococcal carriage among community-dwelling adults in Portugal between 2021 and 2022, during the COVID-19 pandemic. Pneumococcal detection was conducted using molecular methods, with positive samples further characterized by molecular serotyping. Risk factors associated with pneumococcal colonization were also assessed.

We found that the prevalence of pneumococcal carriage among Portuguese adults was 7%, which is lower than in our previous studies on healthy adults aged 25–50 years (cumulative incidence of 28.7%) and adults over 60 years (10%) (15, 16). Direct comparisons with our previous studies should not be done due to methodological differences, different target populations, and the exceptional timing (COVID-19 pandemic) of the current study which affected mixing patterns (15, 16).

The carriage rate was also lower than rates reported in studies from the Netherlands (17, 18, 20, 21) and the United States (22, 23), where it ranged from 16% to 40% using similar molecular methods. Differences in these findings may be attributed to variations in sampling methodologies, population characteristics, and transmission patterns. In the current study, carriage was assessed directly from swabs preserved in a transport inactivation medium suitable for nucleic acid extraction but not for culture-enrichment, a step known to enhance pneumococcal detection by reducing the presence of other microorganisms (17, 21, 24). In contrast, the abovementioned studies used culture enrichment and, in some cases, saliva samples, which could account for the higher sensitivity reported (18, 20).

This study was conducted during the COVID-19 pandemic, a period marked by the implementation of non-pharmaceutical interventions such as social distancing, mandatory mask use, and school closures. From February 2021 to February 2022, when this study was conducted, Portugal implemented various COVID-19 restrictions in response to different waves of the pandemic. Between January and mid-March 2021, the country was under full lockdown, with schools closed and only essential services operating. Remote work was mandatory, and mask use was required in all indoor and outdoor public spaces. Starting 15 March 2021, restrictions were progressively lifted, with schools and services reopening in phases, although masks remained mandatory. From September 2021 onwards, the use of masks was no longer required in outdoor settings. With the emergence of the Omicron variant in December 2021, Portugal reinstated a State of Calamity. During this period, remote work was recommended, and mandatory mask use was reintroduced in indoor public spaces. Schools remained open, although their reopening after the Christmas holidays was delayed by one week. By February 2022, most restrictions had been lifted, with mask use remaining mandatory only in indoor settings (25). These measures likely reduced pneumococcal transmission, contributing to the low prevalence observed. Similar impacts have been reported elsewhere, such as in Denmark, where a decrease in pneumococcal carriage among senior adults during lockdown was noted, although the prevalence rebounded to pre-lockdown levels after restrictions were lifted (25). However, other studies, including those conducted among adults in the United States (26) and among children in France, Belgium, and Israel, found no significant changes in pneumococcal carriage rates during the pandemic (27–29). The variable findings among these studies may be due to several factors, such as differing recommendations and levels of adherence to non-pharmacological interventions, including school attendance, social distancing, and mask use.

Consistent with prior research, we observed that combining nasopharyngeal and oropharyngeal samples significantly improved the detection of pneumococcal carriage (15–18, 21), with a 1.9-fold increase compared to nasopharyngeal samples alone and a 1.3-fold increase compared to oropharyngeal samples alone. Additionally, the density of pneumococci was higher in nasopharyngeal samples than in oropharyngeal samples, as reported in previous studies (15, 16).

The theoretical coverage of pneumococcal vaccines varied widely, with PCV21 providing the highest potential coverage (64.1%), followed by PCV20 (40.0%). The low estimated coverage of PCV13 (13.5%) reflects its widespread use among Portuguese children and the resulting herd effect. Indeed, vaccination uptake for PCV13 in Portuguese children is exceptionally high, being 98.9% in 2021 (7). Specific vaccination rates among adults are unavailable but are expected to be significantly lower; in our study, only 2.5% of adults reported receiving a pneumococcal vaccination. Of note, the exclusion of serotype 5 due to concerns about false positives may have led to an underestimation of PCV13, PCV15, and PCV20 coverage. We note, however, that in carriage studies conducted in 1,450 children in 2018–2020, where pneumococci were isolated and individually serotyped, serotype 5 was never detected (30).

Beyond reducing disease and carriage rates through herd immunity, recent evidence suggests that PCV13 elicits functional antibodies that disrupt pneumococcal biofilms formed by vaccine serotypes (31). Biofilm formation is the primary mode of *S. pneumoniae* colonization, enhancing bacterial persistence and immune evasion. Their disruption by PCV13-induced antibodies provides an additional mechanism by which vaccination can reduce transmission. These findings further support the role of expanded-valency PCVs in decreasing the burden of pneumococcal carriage and disease in adults.

Carriage of PCV13 serotypes was mainly driven by serotype 3, detected in 6.9% of carriers. This serotype is associated with both invasive and non-invasive disease in adults in Portugal, accounting for 15% of adult non-invasive pneumococcal pneumonia cases between 2016 and 2018, and 17% of adult invasive disease cases from 2019 to 2023 (32, 33). Other PCV13 serotypes, such as 19F and 19A, which remain a significant cause of disease in adults (34, 35), were rare, accounting for less than 1% of carriers.

In contrast to recent findings from other studies, we observed a very low prevalence of serotype 4 in our population, with only one carrier identified. Serotype 4 has been increasingly reported in invasive disease cases among adults (18–64 years) in several European countries and the United States (32, 33, 36). A recent study in Portugal highlighted a significant rise in serotype 4 cases among adults, accounting for 4% of invasive disease cases (34), although it did not contribute to invasive disease among children (37). The epidemiology of serotype 4 can vary by region, and its very low prevalence in our carriage study may be explained by several factors. One possibility is that serotype replacement dynamics differ between high-income countries, influencing serotype circulation patterns (38). Additionally, serotype 4 may be circulating at very low levels in Portugal, making detection challenging. Local transmission dynamics, the effects of vaccination programs, and the inherent lower propensity of serotype 4 to persist as a colonizer in healthy adults could further explain the observed discrepancy (39). Further surveillance studies are needed to clarify the role of serotype 4 in pneumococcal carriage and transmission in Portugal and to assess its potential re-emergence in both carriage and disease.

Among non-PCV13 serotypes, serotypes 8, 10A, 11A/D, 16F, 23B, 31, and 33A/F/37 were most frequent, collectively accounting for 43% of detected serotypes. Many of these serotypes are targeted by PCV21, with some also covered by PCV20. Notably, serotype 8, a major cause of invasive disease in adults in Portugal in 2019–2023, and other European countries, was relatively frequent, accounting for 4.9% of detected serotypes (34, 40–44). A high prevalence of serotypes 11A/D, 23B, and NT has also been observed in carriage among children in Portugal since 2015 (10, 30). Similarly, serotypes 10A, 23B, and 11A/D were among the most frequent serotypes carried by adults in the United Kingdom and the United States in recent studies (45–47). Additionally, serotype 23B has been significantly associated with invasive disease, while serotypes 11A and 23B have been associated with non-invasive pneumococcal disease among adults in Portugal during comparable periods (34, 35).

Contact with children under 18 years increased the risk of pneumococcal carriage nearly threefold, consistent with findings that children are significant reservoirs and transmitters of pneumococci (16, 18, 26, 48). Of note, recent studies suggest that adults may also play a role in transmitting pneumococci (16, 45), a topic currently under investigation.

Being male was associated with a lower risk of colonization. While this observation aligns with studies reporting sex differences in susceptibility to respiratory infections (49, 50), it contrasts with earlier findings linking male sex to a higher risk of pneumococcal carriage (51, 52). This discrepancy may be influenced by cultural factors. In Portugal, the majority of civil servants in roles such as preschool and nursing home assistants are women (53). These occupations may lead to increased exposure to pneumococci, potentially explaining the observed difference (54, 55).

A previous study from our group identified smoking as a significant risk factor for pneumococcal carriage (12). That study, conducted between 2010 and 2012, focused on adults aged ≥60 years living in both urban and rural areas of Portugal, with a median age of 75 years (12). However, this association was not observed in our current study, nor in another study we conducted in 2015–2016 among adults aged 25–50 years living in an urban area (16). One possible explanation for this discrepancy could be differences in population composition, sample size, and methodological approaches. Differences in geographical context, changes in smoking trends over time, and potential interactions with other unexamined risk factors may also have contributed to this variation.

This study has some limitations. It relied on a convenience sample of civil servants in Oeiras, which may not represent the broader Portuguese population. The relatively low prevalence of pneumococcal carriage may have limited the statistical power to identify additional risk factors. Furthermore, the use of an inactivation medium precluded culture-enrichment and subsequent genotyping or antimicrobial susceptibility testing of strains. It also precluded the assignment of a specific single serotype in cases where currently available primers and probes do not allow such discrimination (e.g., 33A/33F/37). Another limitation was the lower specificity of qPCR assays for certain serotypes, potentially due to cross-reactivity with non-pneumococcal strains, particularly in oropharyngeal samples. To address this, we excluded results from sites where false positives were detected, ensuring confidence in our findings. Additionally, the inability to assign specific serotypes to certain capsular types or to determine serotypes in a quarter of pneumococcal carriers represents another constraint, though the primers and probes used targeted most serotypes reported in recent surveillance studies in Portugal (10, 12, 16, 30).

Despite these limitations, this study has several strengths. It was a prospective investigation involving over 3,500 paired nasopharyngeal and oropharyngeal samples. The use of qPCR targeting multiple probes enhanced the detection of pneumococci compared to culture-based methods. We also performed rigorous analyses of molecular serotyping and potential false-positive results, while addressing multiple colonization, which is critical for evaluating vaccine impacts and serotype replacement.

In conclusion, this study on pneumococcal carriage among Portuguese adults highlights the circulation of serotype 3 alongside other epidemiologically relevant serotypes not targeted by PCV13. Contact with children was identified as a significant risk factor for carriage. The findings underscore the high potential coverage of novel vaccines with expanded valency, which are critical for adult immunization programs. These data establish a baseline for monitoring vaccine impacts on pneumococcal colonization and provide valuable insights for public health strategies aimed at reducing pneumococcal disease. From a public health perspective, our results challenge the traditional view of pneumococcal carriage as predominantly child-focused, highlighting the interconnected roles of adults and children in transmission dynamics. This emphasizes the need for comprehensive prevention strategies that include adult vaccination and community-level interventions to effectively mitigate pneumococcal disease.

## MATERIALS AND METHODS

### Study design and population

This was an observational prospective study. Civil servants aged over 18 years old and working at the Municipality of Oeiras, Portugal, were enrolled between February 2021 and February 2022 during routine testing for asymptomatic carriage of SARS-CoV-2. For each participant, one nasopharyngeal and one oropharyngeal sample were obtained.

Civil servants were selected for this study as a convenience sample, as they were regularly involved in routine SARS-CoV-2 testing, providing an accessible study population. While this cohort may not be fully representative of the general Portuguese adult population, it includes individuals from diverse professional backgrounds, encompassing both office-based and fieldwork roles, such as urban cleaning services employees, gardeners, nursing home employees, school assistants, day-care center assistants, firefighters, police officers, and summer camp monitors, as well as other professionals and retired individuals. This diversity ensured a range of occupational exposures. Given their roles in the community, several of these participants continued working at their usual locations during the COVID-19 pandemic, despite restrictions on movement.

Civil servants were invited to donate the samples for research purposes and to fill in a questionnaire with demographic and clinical information relevant for the study of risk factors associated with carriage of pneumococci and infection by SARS-CoV-2.

### Sampling and transport

Sampling was performed by nurses using a flexible swab with a flocked nylon fiber tip (ESwab 482CE, Copan) to transnasally reach the nasopharynx, and using a rigid swab with a flocked nylon fiber tip (ESwab 480CE, Copan) to transorally reach the oropharynx. Swabs were placed in an in-house viral inactivation medium containing guanidinium thiocyanate, kept at 4°C, and transported within 4 h to the laboratory. Upon arrival to the laboratory, aliquots of each sample were prepared and kept at −80°C until further use.

### Detection of *S. pneumoniae*

Nasopharyngeal and oropharyngeal samples were thawed on ice, vortexed for 20 s and 200 μL were transferred into a tube with 200 μL of a lysis buffer (MagNA Pure Compact Nucleic Acid Isolation Kit, Roche Diagnostics GmbH), and incubated for 20 min at 37°C. Total DNA was then extracted using the MagNA Pure Compact Instrument (Roche Diagnostics GmbH) as recommended by the manufacturer.

*S. pneumoniae* carriage was evaluated by real-time PCR (qPCR), using specific primers and probes targeting two pneumococcal genes: *lytA* (56) and *piaB* (17) as previously described (15–17). Samples were considered positive for pneumococci when cycle threshold (Ct) values for both genes were below 40 and there was agreement between the Cts (defined as not differing from each other in more than 2 Cts) obtained for each gene (15–18). For samples exhibiting unusual results, such as the absence of *piaB* or a difference of Ct values between *lytA* and *piaB* outside the defined concordance limits, an additional qPCR targeting SP2020 was also performed (57). In those cases, samples were considered positive if the Ct value for SP2020 was below 40 and the Cts obtained for *lytA* and SP2020 did not differ from each other in more than 2 Cts (57).

### Capsular typing

*S. pneumoniae* serotyping was determined by uniplex qPCR following a pooling strategy as previously described (18). Briefly, pools of five pneumococcal positive samples were tested; if a Ct < 40 was obtained for a given pool, the individual samples were tested to identify the serotype that originated the signal. Samples were considered positive for a given serotype or serogroup if the Ct value was below 40 and the Ct serotype was less than or equal to CtlytA or CtpiaB plus 2, indicating a comparable or lower density of the serotype-specific target compared to the lytA- or piaB-specific targets (16, 19).

A total of 66 serotypes, including all serotypes targeted by one or more of the following vaccines—PCV13 (Pfizer), PCV15 (Merck Sharp & Dohme [MSD]), PCV20 (Pfizer), PCV21 (MSD), and PPV23 (MSD)—were identified using primers and probes described in previous studies with the exception of serotypes 4 and 24B/F, which primers are described here (Table S1, Supplemental text) (58–60). In some cases, additional primers were used to discriminate specific serotypes within a group (6A/B, 6B/D, 6C/D, 10A, 10B, and 22F) (Table S1) (60, 61). A sample was considered uncapsulated and designated non-typeable (NT) if it was positive for *lytA* and SP2020 genes (with concordant Cts), but negative for *piaB,* and no capsular type assignment could be obtained (19).

In addition, to detect potential false-positive results, a random sample of pneumococcal negative samples (300 nasopharyngeal and 300 oropharyngeal samples) was also tested by qPCR for all serotypes/serogroups yielding positive results in pneumococcal positive samples. To streamline the process, pools of ten pneumococcal negative samples, grouped by sampling site, were tested.

To estimate the potential coverage of PCVs, serotype 6C was included with PCV13 serotypes, as the 6A antigen has been shown to provide cross-protection against 6C (62).

### Statistical analysis

Socio-demographic and clinical variables included in the questionnaire were summarized using the mean and standard deviation or percentages, as appropriate. The Fisher’s exact test was used to compare categorical variables, and the Student’s *t*-test was employed to compare the average age between the two pneumococcal strata. Population characteristics that were significantly different at a *P* value of 0.1 were selected for downstream analysis. These were further explored using a multivariate GLM with a logit link function, and the adjusted ORs were calculated by exponentiating the model coefficients. Since significant differences between crude ORs and adjusted ORs can indicate the presence of confounding, both were estimated for comparison. It is well established that multicollinearity in a model can inflate the variance of regression coefficients. To address this, severe multicollinearity in the GLM was assessed by calculating the variance inflation factor (VIF) and ensuring it was below 5 (63). Additionally, the assumption of linearity between independent variables and log odds was verified using the Hosmer-Lemeshow test (64). Given the unbalanced number of observations between pneumococcal carriers and non-carriers (the response) and the limited number of observations at certain covariate levels, the chosen model could introduce bias in OR estimation due to its reliance on maximum likelihood estimation. This bias, known as sparse data bias, can result in disproportionately large OR estimates (65). To address this problem, the methods proposed by Gosho et al. (65) were employed, including Bayesian estimation via a posterior distribution. This was implemented using adaptive sampling without replacement for variable selection in GLMs, with the BAS package in R (66). The prior distribution for the coefficients was based on the class of g-priors corresponding to the “test-based Bayes factors,” while a uniform distribution was used as the prior for the models. The sampling method employed was a Markov chain Monte Carlo algorithm that combined a random walk Metropolis-Hastings approach with a random swap of currently included variables with excluded ones. Model averaging and variable selection were performed across 10,000 iterations for all possible models with up to 2^no. of variables^.

The geometric mean and standard deviation were used to summarize the distribution of Ct values from *lytA* and *piaB* in nasopharyngeal and oropharyngeal samples. To access associations between Ct values from nasopharynx versus oropharynx, an adjusted GLM with a Gaussian distribution and a log link function was used.

The McNemar’s test was applied to compare the prevalence of *S. pneumoniae* between nasopharyngeal and oropharyngeal samples. In addition, we used the Cochran’s *Q*-test to compare paired prevalences of pneumococci between nasopharynx, oropharynx, and both. The GSID and the percentile bootstrap method were used to calculate serotype diversity in each sampling site and the 95% CI, respectively. A *P* value of < 0.05 was considered statistically significant. All statistical analyses were conducted using R v4.3.1 (67).

## SUPPLEMENTAL MATERIAL

Supplemental material msphere.00082-25-s0001.pdf Supplemental text, tables, and figures.

ASM does not own the copyrights to Supplemental Material that may be linked to, or accessed through, an article. The authors have granted ASM a non-exclusive, world-wide license to publish the Supplemental Material files. Please contact the corresponding author directly for reuse.
