# Supplementary material for: Streptococcus pneumoniae carriage in adults during the COVID-19 pandemic in Portugal: dominance of serotypes included in broader PCVs and of serotype 3
Source: mSphere. 2025 Jun 10;10(7):e00082-25. doi: 10.1128/msphere.00082-25 (PMC12306169; doi:10.1128/msphere.00082-25)
Supplement: Supplemental material — Supplemental text, tables, and figures. [file msphere.00082-25-s0001.pdf]

**SUPPLEMENTARY MATERIAL**

**Design and validation of specific primers and probes for accurate detection of serotypes 4 and 24B/F**

We found that primers and probes previously described for 4 and 24A/B/F (1, 2) produced false positive results (Supplementary Table 1). Given that serotypes 4 and 24F are targeted by PCV13 and PCV21, respectively, we designed novel primers and probes to improve specificity. To do so, we analyzed the DNA sequences of capsular operons of serogroup 24 of *S. pneumoniae* strains from a collection of pediatric carriage isolates (3), a reference 24F strain (4), and *S. mitis* isolates carrying 24F capsule sequences (5). For serotype 4, we followed the same approach, analyzing the DNA sequences of capsular operons from a reference serotype 4 strain (4) and non-pneumococcal strains with serotype 4 capsule sequences (6). Sequence alignments were performed using Qiagen CLC Genomics Workbench software v9.5.1 (Qiagen, Venlo, The Netherlands). Specific primers and FAM-labeled probes were designed to detect serotypes 4 and 24B/F capsules of *S. pneumoniae* by amplifying target sequences of 218-bp and 150-bp, respectively:

Serotype 4:
4_F (5'-GGTGAAAAATTCTTTAATGAATATCG-3')
4_R (5'-GTCATTTGCAAAAAAATCG-3')
4_P (5'-Fam-CTCGTTTAATTCTATCATATTGGGT-MGB-3')

Serogroup 24:
24B/F_F (5'-TAGGGAAAATAAAATGTTGGCAGC-3'),
24B/F_R (5'-GTTAGAGTAATCATCCACAGTC-3'), and
24B/F_P (5'-Fam-AAAATTGTCAGCAACCAATAAATTAC-BHQ1-3').

The reaction mixtures were performed in a final volume of 25µL containing 1X FastStart TaqMan Probe Master (Roche), 300 nM of each primer, and 200nM of probe for serotype 4; and 200nM of each primer, and 100nM of probe for serogroup 24. DNA was amplified with the CFX96 Real-Time System Amplification (Bio-Rad) by using the following cycling conditions: 95°C for 10 min followed by 40 cycles of 95°C for 15 sec and 52°C for 2 min 15seg for serotype 4, and 95°C for 10 min followed by 40 cycles of 95°C for 15 sec and 55°C for 1 min for serogroup 24.

Assay specificity was validated using: (i) pure cultures of serotype 4 (n=2) and of serotype 24F *S. pneumoniae* (n=12), previously characterized and confirmed by whole genome sequencing; and (ii) pools of samples (n=60, 30 nasopharyngeal pools of 10 samples each and 30 oropharyngeal pools of 10 samples each) confirmed to be negative for pneumococci (*lytA* and *piaB* negative). Previously

described primers (1, 2) gave positive results in one nasopharyngeal pool and 17 oropharyngeal pools for serotype 4, and in three nasopharyngeal pools and 23 oropharyngeal pools for serogroup 24.

With the newly designed oligonucleotides, all serotype 4 and 24F pure cultures yielded positive results. Importantly, all 60 pools of pneumococcal negative samples were negative for serotypes 4 and 24B/F when the respective new primers and probes were tested.

**Supplementary Table 1. Primers and probes used in this study for pneumococcal serotyping deduction by qPCR**

| Serotype/ Serogroup | Primers/Probe | Sequence (5’- 3’) | Reference | False positive results obtained in this collection[1] | False positive results obtained in other studies using these primers and probes |
|---|---|---|---|---|---|
| 1 | 1-F<br>1-R<br>1-P | TTTCATCCCTATGTGTGGTATAG<br>GCTTTAGAAGGTAGAGTTAACAAC<br>FAM-TGCCAAAGCCAGCCAT-BHQ1 | (1) | 0 | _ |
| 2 | 2-F<br>2-R<br>2-P | TGTTATCCCATATAAGAACCGAGTGT<br>AAAATTACCCCAAAAGCTATCCAA<br>HEX-TTGCAATT”T”CAATTTTTTTGCCCCAATCTC-BHQ1 | (1) | 0 | _ |
| 3 | 3-F<br>3-R<br>3-P | CCACTAAAGCTTTGGCAAAAGAAA<br>CCCGAACGTAAAGCTTCTTCA<br>HEX-TTGTAGACCGCCCCACAA”T”TCATTTTGT-BHQ1 | (1) | 0 | _ |
| 4 | 4-F<br>4-R<br>4-P | GCTTCTGCTGTAACTGTTGTGC<br>CACCACCATAGTAACCAAAGTTCC<br>CY5-TTCCACAAAAGAAGAGCCTACAGGTAACCCCA-BHQ3 | (1) | NP samples (1 pool)<br>OP samples (17 pools) | NP, OP, and saliva samples from adults (7)<br>NP samples from children (8) |
| 4 | 4-F<br>4-R<br>4-P | GGTGAAAAATTCTTTAATGAATATCG<br>GTCATTTGCAAAAAAATCG<br>FAM-CTCGTTTAATTCTATCATATTGGGT-MGB | This study | 0 | _ |
| 5 | 5-F<br>5-R<br>5-P | CATGATTTATGCCCTCTTGCAA<br>GACAGTATAAGAAAAAGCAAGGGCTAA<br>FAM-TCTTCTTCTCA”T”CGTTTCCGCATGCTTTTBHQ1 | (1) | NP samples (1 pool)<br>OP samples (11 pools) | NP, OP, and saliva samples from adults (7)<br>NP samples from children (8) |
| 6A/B/C/D | 6A/B/C/D-F<br>6A/B/C/D-R<br>6A/B/C/D-P | GTTTGCACTAGAGTATGGGAAGG<br>TAGCCTTTCTGAAAACATTTAGCG<br>FAM-TGTTCTGCCCXTGAGCAACTGGTCTTGTATC-BHQ1 | (1) | 0 | _ |
| 6A/B | 6A/B-F<br>6A/B-R<br>6A/B-P | CTGATAAAGTTTCGGATAGAAATAA<br>AACGTTCTCTATCCAATTAATTTCTC<br>CY5-AGAAAAGATAAATAGATTATCAAAACAATTTGCGCAGA-BHQ3 | (2) | 0 | – |
| 6C/D | 6C/D-F<br>6C/D-R<br>6C/D-P | TTGGGATGATTGGTCGTATTAG<br>CTCTTCAATTAGTTCTTCAGTTCG<br>CY5-CCACGCAATTCGCCATC-BHQ1 | (2) | 0 | _ |
| 6B/D | 6B/D-F<br>6B/D-R<br>6B/D-P | GCATTGCTAGAGATGGTTCCT<br>CGATACAAGACCAGTTGCTCA<br><HEX>A<pdC><pdU>G<pdU><pdC><pdU><pdC>A <pdU>GA<pdU>A A<pdU><pdU>A<pdU><pdU> <BHQ1> | (2) | 0 | – |
| 7A/F | 7A/F-F<br>7A/F-R<br>7A/F-P | ATGAAGGCTTTGGTTTGACAGG<br>ATTCTCGCCATCAATTGCATATTC<br>CY5-ACACCACTATAGGCTGTTGAGACTAACGCACA-BHQ3 | (1) | 0 | _ |

**Supplementary Table 1.** *(cont.)*

| Serotype/ Serogroup | Primers/Probe | Sequence (5'- 3') | Reference | False positive results obtained in this collection[1] | False positive results obtained in other studies using these primers and probes |
|---|---|---|---|---|---|
| 7C/B1 | 7C/B1-F<br>7C/B1-R<br>7C/B1-P | GTGAAAAAAAGTAGTACGTTACATAG<br>GGTACTAAATTAAAGAAGTTTTTACTCA<br>HEX-AGTACGTTACATATAGGACTTATTCTTTTTTTGATTGT-BHQ1 | (2) | 0 | _ |
| 7C/B2 | 7C/B2-F<br>7C/B2-R<br>7C/B2-P | TTGAGCATAACGGAGCGATA<br>AGCAGCTATATCATAAGCAATCG<br>FAM-TGTTCCGAATATTGGTCCAGCTCGAG-BHQ1 | (2) | 0 | _ |
| 8 | 8-F<br>8-R<br>8-P | CCACTCATCAGTTTCCCATATGTTT<br>TCAATAATTGAAGAAGCGAACGTT<br>FAM-TGATGGCAGATGGGTTGGGACGAG-BHQ1 | (9) | 0 | _ |
| 9A/V | 9A/V-F<br>9A/V-R<br>9A/V-P | AGGTATCCTATATACTGCTTTAGG<br>CGAATCTGCCAATATCTGAAAG<br>HEX-ACACATTGACAACCGCT-BHQ1 | (1) | 0 | NP, OP, and saliva samples from adults (7) |
| 9N/L | 9L/N F<br>9L/N-R<br>9L/N-P | CGTGGAATTTTCTATACTGCAATAGG<br>CTACTGCTACGATACCATATTCTACAG<br>CY5-CAGCAATTCTTAGCCGGATTCTCTCAC-BHQ3 | (2) | OP samples (8 pools) | _ |
| 10A/B | 10A/B-F<br>10A/B-R<br>10A/B-P | CCTCTCCTATCAACTATTACTCATTATACTACCT<br>AATAACCATAAGTCCCTAGATCATTCAAAG<br>FAM-TCATTACAACTCCCTATGTGACACGGGTCTTTT-BHQ1 | (9) | 0 | _ |
| 10A | 10A-F<br>10A-R<br>10A-P | TAGTGTCGGCAGACAAATTAT<br>CACGCTCATACACTTTATTTGA<br>CY5-TTGAGCATGGTCTCTGATGAGATTT-BHQ3 | (2) | 0 | _ |
| 10B | 10B-F<br>10B-R<br>10B-P | TGCAGAGATGTTAGGATTCCGT<br>TCGGCCTGCACTAATGATAAA<br>FAM-TGTTCCATTTGTTGTCAGGTG-MGB | (10) | 0 | _ |
| 11A/D | 11A/D-F<br>11A/D-R<br>11A/D-P | AAATGGTTTGGATATGGTTTGTTTGG<br>AGTGCTAACTGTAAAACTTGATTATGAG<br>CY5-ATTCCAACTTCTCCCAATTTCTGCCACGG-BHQ3 | (1) | 0 | _ |
| 11B/C | 11B/C-F<br>11B/C-R<br>11B/C-P | CCGCTATCAAATTTGGCGTATTG<br>AGCTGATTATGAGCATAGTTGATCC<br>HEX-TCCGTGGCAAGATTCTGGTGCTAG-BHQ1 | (2) | NP samples (1 pool)<br>OP samples (16 pools) | _ |
| 12A/B/F/44/46 | 12A/B/F/44/46-F<br>12A/B/F/44/46-R<br>12A/B/F/44/46-P | GCACCCACGGGTAAATATTCTAC<br>CAACTAAGAACCAAGGATCCACAG<br>CY5-TGCCCACCAACACCAGGTCCAGGT-BHQ3 | (1) | 0 | _ |
| 13 | 13-F<br>13-R<br>13-P | AGACTACCATTTTTTGATCAGTTAGATT<br>CAGAAAACATATTTTGTTCATAAATCCATC<br>FAM-AAGCAGCACTTCCAAGTCGTAATCTACC-BHQ1 | (2) | 0 | _ |

**Supplementary Table 1.** *(cont.)*

| Serotype/ Serogroup | Primers/Probe | Sequence (5’- 3’) | Reference | False positive results obtained in this collection[1] | False positive results obtained in other studies using these primers and probes |
|---|---|---|---|---|---|
| 14 | 14-F<br>14-R<br>14-P | AGAGTGTATGAGGAATCC<br>ATATATCTACTGTAGAGGGAAT<br>FAM-CGCCAAGTAACA”T”TTCCATTCCATT-BHQ1 | (1) | 0 | _ |
| 15A/F | 15A/F-F<br>15A/F-R<br>15A/F-P | AAGCAGCACTTCCAAGTCGTAATCTACC<br>ACTTCAATTAATAAGCGGATGATTGTAGCGT<br>FAM-AGCAATATAAGAGGTATAGTTGGATAA-BHQ1 | (1) | 0 | _ |
| 15B/C | 15B/C-F<br>15B/C-R<br>15B/C-P | AAGCAGCACTTCCAAGTCGTAATCTACC<br>ACTTCAATTAATAAGCGGATGATTGTAGCGT<br>FAM-AGCAATATAAGAGGTATAGTTGGATAA-BHQ1 | (2) | 0 | _ |
| 16F | 16F-F<br>16F-R<br>16F-P | TAATGTTATGACCTTGGTAATCTTCCC<br>TCCCAAAGGATAATCAATAACTTTTAGAAG<br>FAM-AGCCATAAGTCT”T”CCAAATGCTTAACCGCT-BHQ1 | (1) | 0 | _ |
| 17F | 17F-F<br>17F-R<br>17F-P | CGGAATATCATGGAGCCTATTA<br>AACGTTCTAATTTGTCCACATC<br>TGTTTGCTGATCAGGATGATATCTGG | (2) | OP samples (2 pools) | _ |
| 18A/B/C/F | 18A/B/C/F-F<br>18A/B/C/F-R<br>18A/B/C/F-P | TCGATGGCTAGAACAGATTTATGG<br>CCATTGTCCCTGTAAGACCATTG<br>HEX-AGGGAGTTGAATCAACCTATAATTTCGCCCC-BHQ1 | (1) | 0 | _ |
| 19A | 19A-F<br>19A-R<br>19A-P | CGCCTAGTCTAAATACCA<br>GAGGTCAACTATAATAGTAAGAG<br>FAM-TATCAATGAGCCGATCCGTCACTT-BHQ1 | (1) | 0 | _ |
| 19F | 19F-F<br>19F-R<br>19F-P | TGAGGTTAAGATTGCTGATCG<br>CACGAATGAGAACTCGAATAAAAG<br>CY5-CGCACTGTCAATTCACCTTC-BHQ3 | (1) | 0 | _ |
| 20 | 20-F<br>20-R<br>20-P | AAAGATACTGGCTGAGGAGCTATCTATT<br>AGTCAAAAGTACTCAACCATTCTGATATATTC<br>FAM-AGGATAAGGTCTACTTTGTGGGAGTTC-BHQ1 | (2) | 0 | _ |
| 21 | 21-F<br>21-R<br>21-P | GGTTTAAATATCGCTCCGGGTAT<br>CAAAAAAAGGGCTTGTAGACGAA<br>CY5-TGTGAATTGGACACGTTATGGAGC-BHQ3 | (2) | 0 | _ |
| 22A/F | 22A/F-F<br>22A/F-R<br>22A/F-P | CTTGGGACTTCTCTATTTGTTATAGG<br>TCCCGAAACCAAATTGCTATCCCTCC<br>FAM-AATATGAGTTACCGCCAACTTT-BHQ1 | (2) | 0 | _ |
| 22F | 22/F-F<br>22/F-R<br>22/F-P | CTTGTCAAGTATGCTGAGGATTTG<br>AGATTTCTCCTGGATATAATGCGAT<br>CY5-ACTCAACAAGCTACAGATGGACATGAAGT-BHQ3 | (2) | 0 | _ |

**Supplementary Table 1.** *(cont.)*

| Serotype/ Serogroup | Primers/Probe | Sequence (5'- 3') | Reference | False positive results obtained in this collection[1] | False positive results obtained in other studies using these primers and probes |
|---|---|---|---|---|---|
| 23A | 23A-F<br>23A-R<br>23A-P | CTCCCCTCCATTACCCATTTGG<br>TGAAGAAAGTGCTGTTTGTGAACC<br>CY5-AGCTAGAAC"T"CCCACACTCCCTACTCCCA-BHQ3 | (1) | 0 | _ |
| 23B | 23B-F<br>23B-R<br>23B-P | TTGAAGAAATTGCTCCAGAAACAT<br>CCAAAAGACTAGCCTCAACCACTAA<br>GACAGCAACGACAATAGTCATCTC | (2) | 0 | _ |
| 23F | 23/F-F<br>23/F-R<br>23/F-P | GACAGCAACGACAATAGTCATCTC<br>TCCATCCCAACCTAACACACTTC<br>CY5-ATTGTGTCCA"T"AACCCTTCGTCGTATTTCCAAAG-BHQ3 | (1) | 0 | _ |
| 24A/B/F | 24A/B/F-F<br>24A/B/F-R<br>24A/B/F-P | GGAGCGGGATATATTTCTTCTAGTC<br>CAAACCTACATCGCTTGGATAAT<br>Cy5-TCTATTGTTACYGGTCCATTAGGACGT-BHQ3 | (2) | NP samples (3 pool)<br>OP samples (23 pools) | _ |
| 24B/F | 24B/F-F<br>24B/F-R<br>24B/F-P | TAGGGAAAATAAAATGTTGGCAGC<br>GTTAGAGTAATCATCCACAGTC<br>FAM-AAAATTGTCAGCAACCAATAAATTAC-BHQ1 | This study | 0 | _ |
| 28F/A | 28F/A-F<br>28F/A-R<br>28F/A-P | TTTAGTTCGTGGAGGTAGACT<br>ACATTCCCAATACCTATAAATAGCC<br>CY5-ACCAATTTCAATTCCAGGAGCGAA-BHQ3 | (2) | 0 | _ |
| 31 | 31-F<br>31-R<br>31-P | AGGTTGGGACAAACCTTGC<br>CGTAAGAGAGCCTTCTCAATAGTC<br>CY5-CCCTTAGTGACATCTGTAATGCTATCTTCT-BHQ3 | (2) | 0 | _ |
| 33A/F/37 | 33A/F/37-F<br>33A/F/37-R<br>33A/F/37-P | GGAACTGGTTCAGCAACTATACG<br>GGTTCTAAGACCGTCTGAAATACC<br>Hex-CCCCAAATAGGAC"T"TTTCTGCCATGCCAAA-BHQ1 | (1) | 0 | _ |
| 34 | 34-F<br>34-R<br>34-P | CGTGGAAGTTTCTCGCAAATAA<br>CACGTAAGAAATAGGAGATATGAAGC<br>Hex-TTTACTGAAGACTTAGTCGGATTGGG-BHQ1 | (2) | 0 | _ |
| 35A | 35A-F<br>35A-R<br>35A-P | TTCCTGATTATGTTGAGATTTGGC<br>AGCGTTGATGGAAGTAATGAATATC<br>Hex-ACCAGAGTTAGACACTATCTTGGTTTCC-BHQ1 | (2) | 0 | _ |
| 35B | 35B-F<br>35B-R<br>35B-P | GAAAGGTATGGAGAAGTTGAGAATG<br>TCCATCTCTATTATTCATATTAAACCCTATTA<br>Hex-ATTCCTTACGTAGAACTGTAAGGGAAGG-BHQ1 | (2) | OP samples (5 pools) | _ |
| 35F/47F | 35F/47F-F<br>35F/47F-R<br>35F/47F-P | GTGGTCGTATATACTTGATGAATAAATCG<br>ACATACAAATTATCAACATACAGATAGGTC<br>FAM-TCCATTCAACTGGTCGTCCGAATAATCC-BHQ1 | (2) | 0 | _ |

**Supplementary Table 1.** *(cont.)*

| Serotype/ Serogroup | Primers/Probe | Sequence (5'- 3') | Reference | False positive results obtained in this collection[1] | False positive results obtained in other studies using these primers and probes |
|---|---|---|---|---|---|
| 38 | 38-F<br>38-R<br>38-P | GTCTTACGTAGAACCTCTCTGGATGA<br>TGGTCCTACAAGCGACATGTG<br>FAM-TTGCCACAGATTTGGAATATTTTGGTCGG-BHQ1 | (9) | 0 | – |
| 39 | 39-F<br>39-R<br>39-P | TGCGCTAAGGTATATTCCGTATTT<br>GACATCAAGTTCCCAAACCAATC<br>CY5-TGATGATGGAGCCTATCATTATAAAGCAGC-BHQ3 | (2) | NP samples (6 pools)<br>OP samples (20 pools) | – |

F: forward primer, R: reverse primer, P: probe, Locked nucleic acid nucleotides are underlined. "T"=BHQ1.

[1]Out of 60 pools of 10 samples each: 30 pools of NP samples and 30 pools of OP samples.

**Supplementary Table 2. Pathologies and respiratory symptoms reported by the participants by pneumococcal carriage status**

| | Overall (N=3,574) n (%) | Pneumococcal carriage | | p-value[1] |
|---|---|---|---|---|
| | | No (N=3,329) n (%) | Yes (N=245) n (%) | |
| **Chronic Diseases** | | | | |
| Asthma | 205 (5.7) | 196 (5.9) | 9 (3.7) | 0.198 |
| Diabetes | 129 (3.6) | 118 (3.5) | 11 (4.5) | 0.475 |
| Cardiovascular disease | 42 (1.2) | 40 (1.2) | 2 (0.8) | >0.999 |
| Renal disease | 9 (0.3) | 9 (0.3) | 0 (0.0) | >0.999 |
| Hypertension | 589 (16.5) | 561 (16.9) | 28 (11.4) | **0.026** |
| COPD | 9 (0.3) | 8 (0.2) | 1 (0.4) | 0.473 |
| Obesity | 10 (0.3) | 10 (0.3) | 0 (0.0) | >0.999 |
| **Respiratory Symptoms** | | | | |
| Cough | 26 (0.7) | 23 (0.7) | 3 (1.2) | 0.419 |
| Shortness of breath | 13 (0.4) | 13 (0.4) | 0 (0.0) | >0.999 |
| Sore throat | 22 (0.6) | 22 (0.7) | 0 (0.0) | 0.397 |
| **Other Symptoms** | | | | |
| Chest pain | 5 (0.1) | 5 (0.2) | 0 (0.0) | >0.999 |
| Diarrhea | 2 (0.1) | 1 (0.03) | 1 (0.4) | 0.132 |
| Fatigue | 14 (0.4) | 13 (0.4) | 1 (0.4) | >0.999 |
| Fever | 10 (0.3) | 10 (0.3) | 0 (0.0) | >0.999 |
| Muscle pain | 1 (0.03) | 1 (0.03) | 0 (0.0) | >0.999 |
| Smell and/or taste loss | 5 (0.1) | 3 (0.1) | 2 (0.8) | **0.041** |

[1]Fisher´s exact test.

**Supplementary Table 3. Geometric mean of the distribution of Ct values for *lytA* and *piaB* genes of oropharyngeal and nasopharyngeal samples**

| | Ct values[1] | |
|---|---|---|
| | ***lytA*** | ***piaB*** |
| Nasopharyngeal samples | 32.29 ± 1.11 | 34.28 ± 1.14 |
| Oropharyngeal samples | 34.59 ± 1.08 | 36.22 ± 1.10 |

[1]geometric mean ± geometric SD

## REFERENCES


1. Pimenta FC, Roundtree A, Soysal A, Bakir M, du Plessis M, Wolter N, von Gottberg A, McGee L, Carvalho MaG, Beall B. 2013. Sequential triplex real-time PCR assay for detecting 21 pneumococcal capsular serotypes that account for a high global disease burden. J Clin Microbiol 51:647-52.
2. Velusamy S, Tran T, Mongkolrattanothai T, Walker H, McGee L, Beall B. 2020. Expanded sequential quadriplex real-time polymerase chain reaction (PCR) for identifying pneumococcal serotypes, penicillin susceptibility, and resistance markers. Diagn Microbiol Infect Dis 97:115037.
3. Candeias C, Almeida ST, Paulo AC, Simões AS, Ferreira B, Cruz AR, Queirós M, Touret T, Brito-Avô A, de Lencastre H, Sá-Leão R. 2024. *Streptococcus pneumoniae* carriage, serotypes, genotypes, and antimicrobial resistance trends among children in Portugal, after introduction of PCV13 in National Immunization Program: A cross-sectional study. Vaccine 42:126219.
4. Bentley SD, Aanensen DM, Mavroidi A, Saunders D, Rabbinowitsch E, Collins M, Donohoe K, Harris D, Murphy L, Quail MA, Samuel G, Skovsted IC, Kaltoft MS, Barrell B, Reeves PR, Parkhill J, Spratt BG. 2006. Genetic analysis of the capsular biosynthetic locus from all 90 pneumococcal serotypes. PLoS Genet 2:e31.
5. Kalizang'oma A, Richard D, Kwambana-Adams B, Coelho J, Broughton K, Pichon B, Hopkins KL, Chalker V, Beleza S, Bentley SD, Chaguza C, Heyderman RS. 2024. Population genomics of *Streptococcus mitis* in UK and Ireland bloodstream infection and infective endocarditis cases. Nat Commun 15:7812.
6. Gertz RE, Pimenta FC, Chochua S, Larson S, Venero AK, Bigogo G, Milucky J, Carvalho MDG, Beall B. 2021. Nonpneumococcal strains recently recovered from carriage specimens and expressing capsular serotypes highly related or identical to pneumococcal serotypes 2, 4, 9A, 13, and 23A. mBio 12.
7. Wyllie AL, Rümke LW, Arp K, Bosch AATM, Bruin JP, Rots NY, Wijmenga-Monsuur AJ, Sanders EAM, Trzciński K. 2016. Molecular surveillance on *Streptococcus pneumoniae* carriage in non-elderly adults; little evidence for pneumococcal circulation independent from the reservoir in children. Sci Rep 6:34888.
8. Wyllie AL, Wijmenga-Monsuur AJ, van Houten MA, Bosch AATM, Groot JA, van Engelsdorp Gastelaars J, Bruin JP, Bogaert D, Rots NY, Sanders EAM, Trzciński K. 2016. Molecular surveillance of nasopharyngeal carriage of *Streptococcus pneumoniae* in children vaccinated with conjugated polysaccharide pneumococcal vaccines. Sci Rep 6:23809.
9. Azzari C, Moriondo M, Indolfi G, Cortimiglia M, Canessa C, Becciolini L, Lippi F, de Martino M, Resti M. 2010. Realtime PCR is more sensitive than multiplex PCR for diagnosis and serotyping in children with culture negative pneumococcal invasive disease. PLoS One 5:e9282.
10. Che J, Chen BH, Xu L, Gao Y, Yue MM, Chen ZM, Zhang MJ, Shao ZJ. 2023. Establishment and modification of ninety-seven pneumococcal serotyping assays based on quantitative real-time polymerase chain reaction. Biomed Environ Sci 36:787-799.

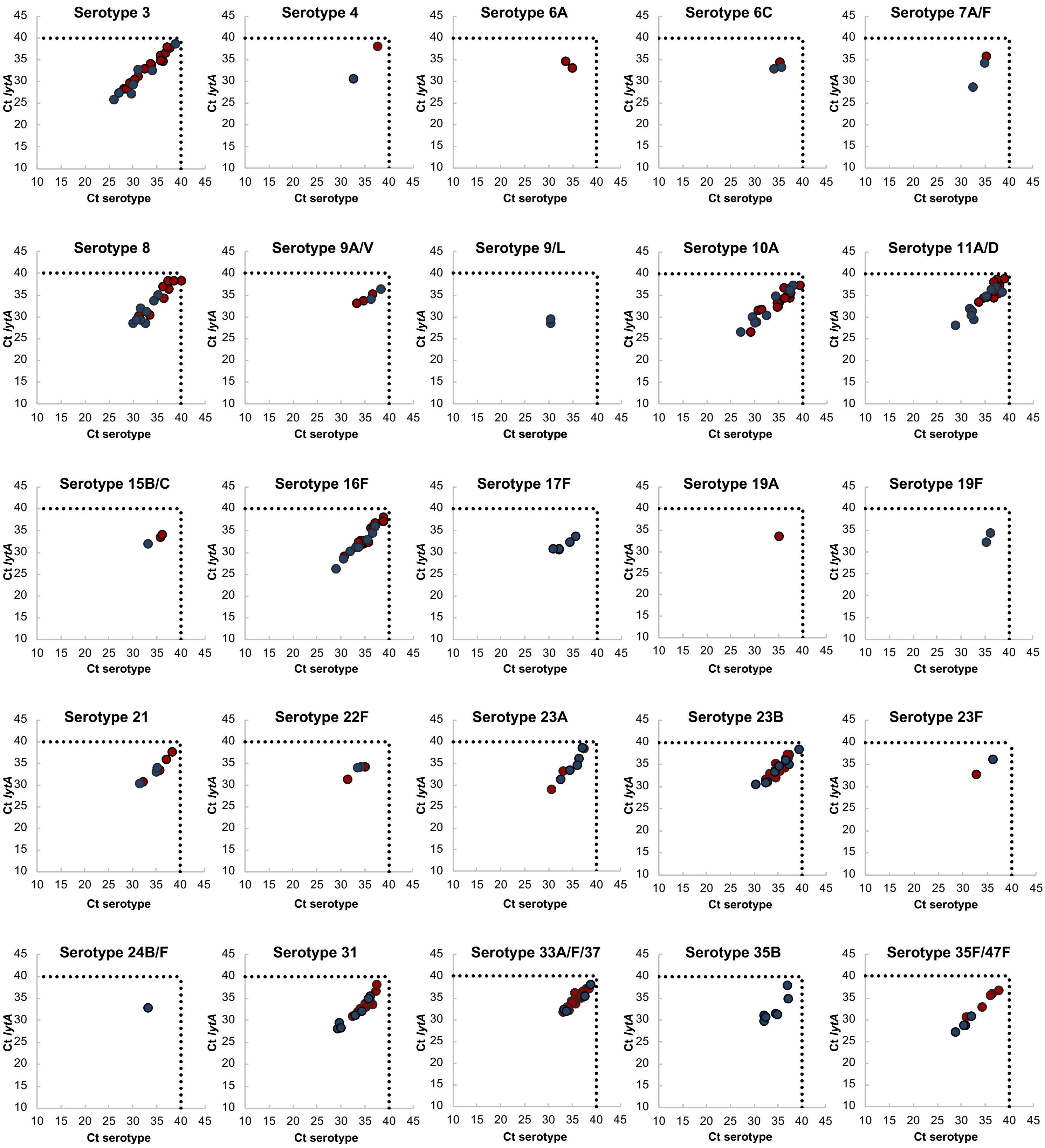


**Supplementary Figure 1**. Graphics show the Ct values obtained for *lytA* and the serotype/serogroup-specific signals in nasopharyngeal and oropharyngeal samples positive for *S. pneumoniae*. Blue circles, nasopharyngeal samples; red circles, oropharyngeal samples. Dashed lines indicate the Ct value of 40, defined to discriminate between positive and negative assays.

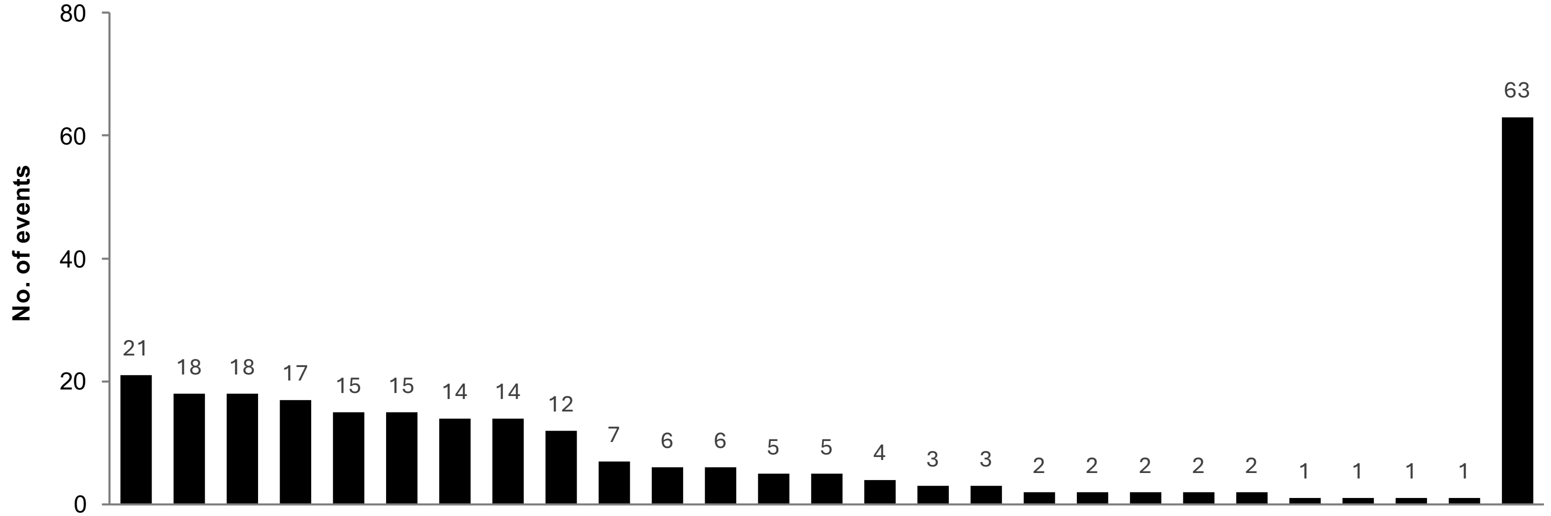


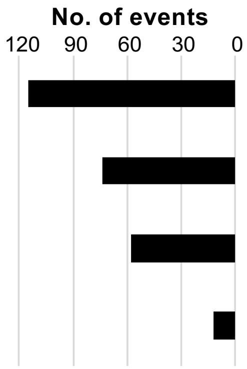


| Carriers (site where Pn was detected) | NT | 10A | 23B | 3 | 11A/D | 33A/F/37 | 16F | 31 | 8 | 35B | 35F/47F | 23A | 17F | 21 | 9A/V | 7A/F | 15B/C | 6A | 6C | 9N/L | 19F | 22F | 4 | 19A | 23F | 24B/F | NA |
|---|---|---|---|---|---|---|---|---|---|---|---|---|---|---|---|---|---|---|---|---|---|---|---|---|---|---|---|
| OP only | 8 | 9 | 10 | 10 | 6 | 11 | 6 | 7 | 3 | NR[1] | 2 | 1 | NR | 2 | 2 | 1 | 2 | 2 | 0 | NR | 0 | 0 | 0 | 1 | 0 | 0 | 32 |
| OP and NP[2] | 6 | 8 | 5 | 4 | 6 | 3 | 7 | 3 | 5 | 0 | 3 | 2 | 0 | 2 | 2 | 1 | 0 | 0 | 1 | 0 | 0 | 2 | 1 | 0 | 1 | 0 | 13 |
| NP only | 7 | 1 | 3 | 3 | 3 | 1 | 1 | 4 | 4 | 3 | 1 | 3 | 5 | 1 | 0 | 1 | 1 | 0 | 1 | 0 | 2 | 0 | 0 | 0 | 0 | 1 | 12 |
| NP with serotype assigned, OP with no serotype assigned[3] | 0 | 0 | 0 | 0 | 0 | 0 | 0 | 0 | 0 | 4 | 0 | 0 | 0 | 0 | 0 | 0 | 0 | 0 | 0 | 2 | 0 | 0 | 0 | 0 | 0 | 0 | 6 |

**Supplementary Figure 2. Distribution of pneumococcal carriage events according to sampling site and serotype distribution.**
[1]NR, non-reliable. Assays targeting these serotypes were classified as unreliable in oropharyngeal samples due to false-positive results. [2]All events had concordant serotypes. Additionally, in two matched OP/NP samples additional serotypes were detected: in one case an additional serotype was detected in the OP sample; in the other case, two additional serotypes were detected in the NP sample. [3]Refers to cases where *S. pneumoniae* was detected in both nasopharynx and oropharynx of the same individual, but a serotype could only be assigned in the NP sample. This was either because the OP sample was negative for all serotypes tested or because the only serotype assigned in the OP sample can give false positive results in pneumococcal OP negative samples. NT, non-encapsulated; NP, nasopharynx; OP, oropharynx; NA, not assigned.
